# Supplementary material for: Sulfonamide Porphyrins as Potent Photosensitizers against Multidrug-Resistant Staphylococcus aureus (MRSA): The Role of Co-Adjuvants
Source: Molecules. 2023 Feb 22;28(5):2067. doi: 10.3390/molecules28052067 (PMC10004250; doi:10.3390/molecules28052067)
Supplement: Supplementary file 1 [file molecules-28-02067-s001.zip › molecules-2220173-supplementary.pdf]

Supplementary Material

Sulfonamide Porphyrins as Potent Photosensitizers against Multidrug-Resistant *Staphylococcus aureus* (MRSA): the Role of Co-Adjuvants

Sofia N. Sarabando [1], Cristina J. Dias [1], Cátia Vieira [2], Maria Bartolomeu [2], Maria G. P. M. S. Neves [1], Adelaide Almeida [2], Carlos J. P. Monteiro [1,*] and Maria Amparo F. Faustino [1,*]

[1] LAQV-Requimte and Department of Chemistry, University of Aveiro, 3810-193 Aveiro, Portugal
[2] CESAM, Department of Biology, University of Aveiro, 3810-193 Aveiro, Portugal
* Correspondence: cmonteiro@ua.pt (C.J.P.M.); faustino@ua.pt (M.A.F.F.)

List of Figures

Figure S1. ^{1}H NMR of **TPP(SO$_2$NHEt)$_4$** in DMSO-d$_6$........ 2

Figure S2. HRMS of **TPP(SO$_2$NHEt)$_4$**........ 3

Figure S3. ^{1}H NMR of **ZnTPP(SO$_2$NHEt)$_4$** in CDCl$_3$/CD$_3$OD(98:2). 3

Figure S4. ^{13}C NMR of **ZnTPP(SO$_2$NHEt)$_4$** in CDCl$_3$/CD$_3$OD(98:2)........ 4

Figure S5. HRMS-ESI of **ZnTPP(SO$_2$NHEt)$_4$**. 4

Figure S6. ^{1}H NMR of **TPP(SO$_3$H)$_4$** in DMSO-d$_6$ (In the main spectrum the expansion of aromatic region is present. Inset the expansion of the shielded porphyrin core inner protons)........ 5

Figure S6. Iodine monitoring formation at 340 nm after irradiation with white light at an irradiance of 25 mW cm^{-2} during 120 min of a solution containing each PS (**TPP(SO$_3$H)$_4$**, **TPP(SO$_2$NHEt)$_4$** and **ZnTPP(SO$_2$NHEt)$_4$**) at a concentration of 5.0 μM and KI at 100 mM. Error bars corresponds to the standard deviation........ 5

Instrumentation and reagents

All the commercial reagents for this work were used directly in all reactions due to their high purity. The evolution of the reactions over the time was motorized by TLC pre-coated with silica gel sheets 60 (Merck). Purifications by column chromatography were performed on silica gel 60. The ^{1}H and ^{13}C NMR spectra were record in a Bruker AMX 300 NMR spectrometer at 300.13 and 75.4 MHz, respectively. The chemical shifts are expressed in δ (ppm) related to TMS as internal reference (δ = 0.00 ppm) and the coupling constants (*J*) in Hertz (Hz). The mass spectra were recorded in an ESI-MS model ionization electrospray spectrometer and HRMS-ESI.
All the solvents used were purified by described methods [1]. The purity of the compounds was confirmed by chromatography, ^{1}H NMR and fluorescence emission spectroscopy.

5,10,15,20-Tetrakis[4-(*N*-ethylsulfamoyl)phenyl]porphyrin (TPP($SO_2NHEt)_4$)

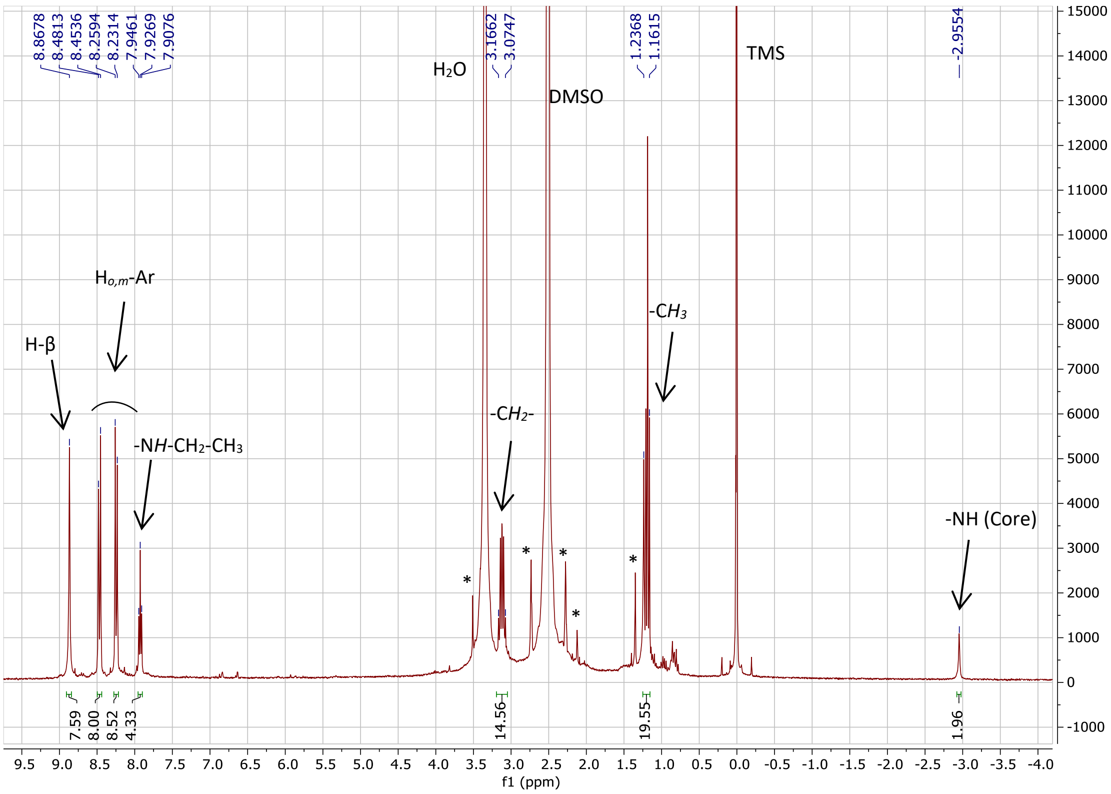

Figure S1. ^{1}H NMR of **TPP($SO_2NHEt)_4$** in DMSO-*d6*. (* Spinning sidebands and/or residual solvents .)

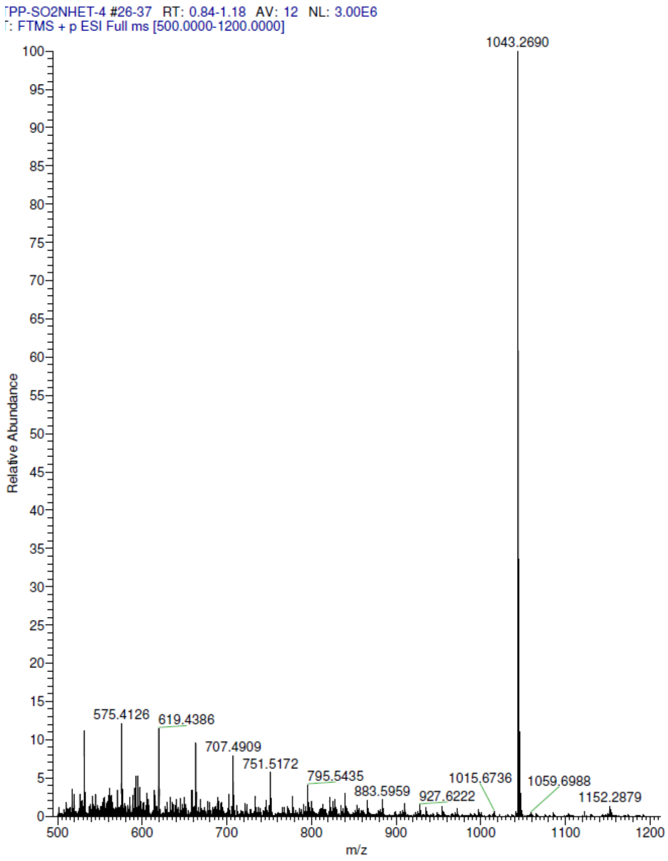

Figure S2. HRMS of **TPP(SO₂NHEt)₄**.

5,10,15,20-Tetrakis[4-(*N*-ethylsulfamoyl)phenyl]porphyrinatezinc(II) (ZnTPP(SO₂NHEt)₄)

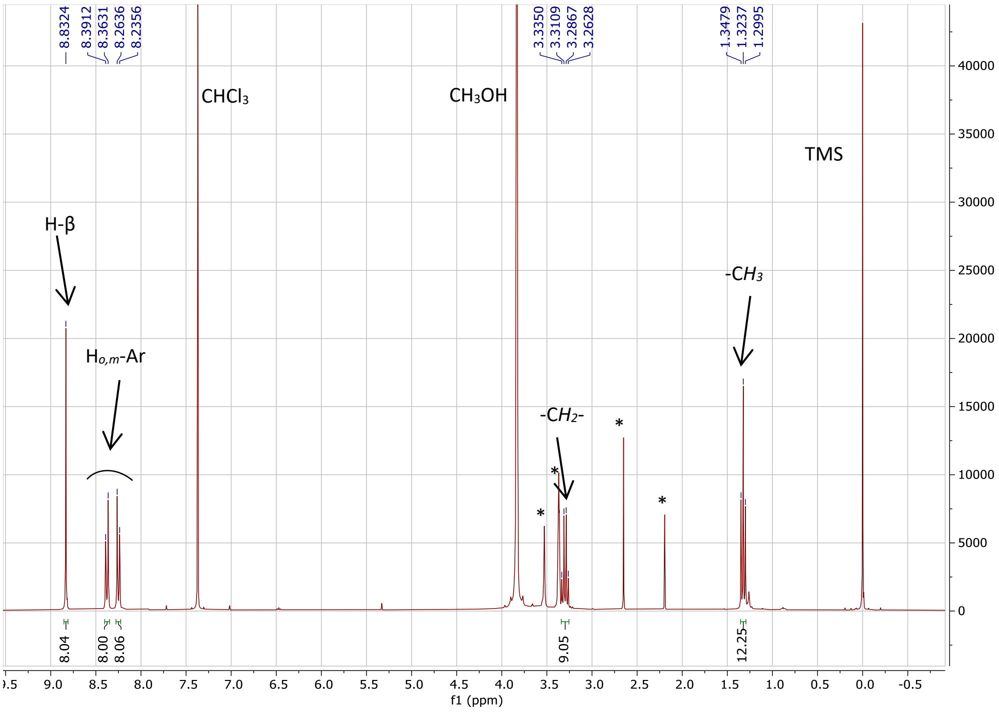

Figure S3. ^{1}H NMR of **ZnTPP(SO₂NHEt)₄** in $CDCl_3/CD_3OD$ (98:2). (* Spinning sidebands and/or residual solvents).

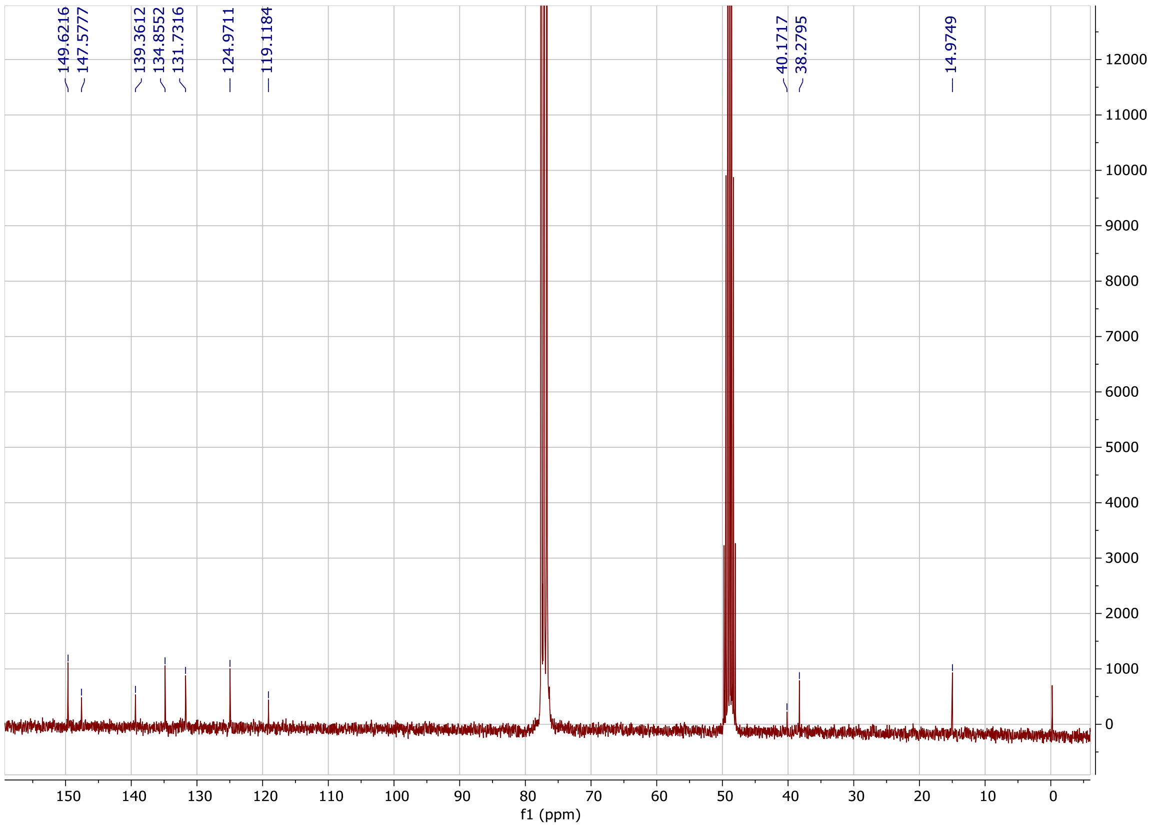

Figure S4. ^{13}C NMR of **$ZnTPP(SO_2NHEt)_4$** in $CDCl_3/CD_3OD$ (98:2).

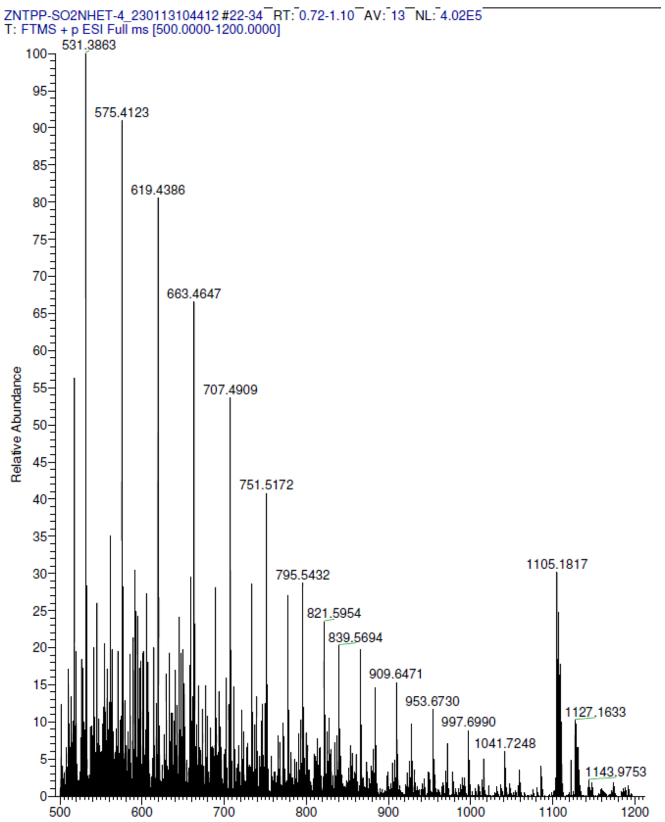

Figure S5. HRMS-ESI of **$ZnTPP(SO_2NHEt)_4$**.

5,10,15,20-tetrakis(4-sulfophenyl)porphyrin, TPP(SO3H)4

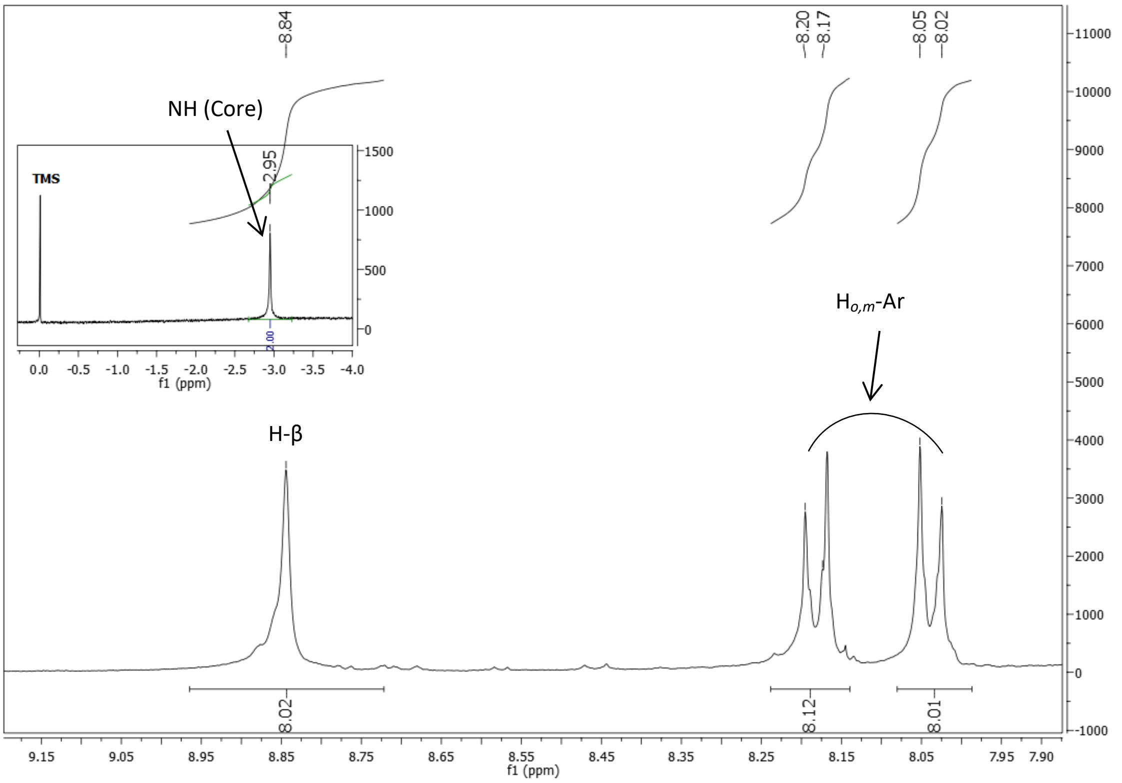

Figure S6. ^{1}H NMR of **TPP(SO3H)4** in DMSO-*d6* (In the main spectrum the expansion of aromatic region is present. Inset the expansion of the shielded porphyrin core inner protons).

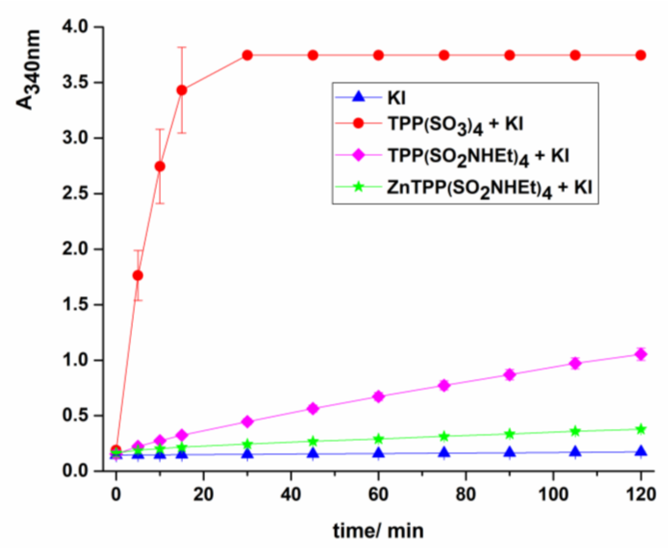

Figure S7. Iodine monitoring formation at 340 nm after irradiation with white light at an irradiance of 25 mW cm^{-2} during 120 min of a solution containing each PS (**TPP(SO3H)4**, **TPP(SO2NHEt)4** and **ZnTPP(SO2NHEt)4**) at a concentration of 5.0 μM and KI at 100 mM. Error bars corresponds to the standard deviation.

Reference

1. Perrin, D.D.; Armarego, W.L.F.; Perrin, D.R. *Purification of Laboratory Chemicals*, 2nd ed.; Pergamon Press: Oxford, **1980**.
